# Modified Rio Score with Platform Therapy Predicts Treatment Success with Fingolimod and Natalizumab in Relapsing-Remitting Multiple Sclerosis Patients

**DOI:** 10.3390/jcm10091830

**Published:** 2021-04-22

**Authors:** Anna Jamroz-Wiśniewska, Radosław Zajdel, Agnieszka Słowik, Monika Marona, Marcin Wnuk, Monika Adamczyk-Sowa, Bożena Adamczyk, Anetta Lasek-Bal, Przemysław Puz, Arkadiusz Stęposz, Ewa Krzystanek, Maja Patalong-Ogiewa, Anna Pokryszko-Dragan, Sławomir Budrewicz, Dorota Koziarska, Anna Karbicka, Sławomir Wawrzyniak, Waldemar Fryze, Marzena Furtak-Niczyporuk, Konrad Rejdak

**Affiliations:** 1 Department of Neurology, Medical University of Lublin, Jaczewskiego 8, 20-054 Lublin, Poland; krejdak@europe.com; 2 Chair of Informatics in Business, University of Lodz, Rewolucji 1905 Roku 37/39, 91-001 Lodz, Poland; radoslaw.zajdel@interforum.pl; 3 Department of Neurology, Collegium Medicum, Jagiellonian University, Jakubowskiego 2, 30-688 Krakow, Poland; agnieszka.slowik@uj.edu.pl (A.S.); monika.marona@interia.pl (M.M.); marcin.a.wnuk@gmail.com (M.W.); 4 Department of Neurology, School of Health Sciences in Zabrze, Medical University of Silesia in Katowice, 3-go Maja 13-15, 41-800 Zabrze, Poland; msowa@sum.edu.pl (M.A.-S.); badamczyk@sum.edu.pl (B.A.); 5 Department of Neurology, School of Health Sciences, Medical University of Silesia in Katowice, Ziolowa 45-47, 40-635 Katowice, Poland; alasek@gcm.pl (A.L.-B.); ppuz@gcm.pl (P.P.); asteposz@gcm.pl (A.S.); 6 Department of Neurology, Faculty of Medical Sciences in Katowice, Medical University of Silesia, Medykow 14, 40-752 Katowice, Poland; ewa26@mp.pl; 7 Department of Neurorehabilitation, Faculty of Medical Sciences in Katowice, Medical University of Silesia, Medykow 14, 40-752 Katowice, Poland; mpatalongogiewa@sum.edu.pl; 8 Department of Neurology, Wroclaw Medical University, Borowska 213, 50-566 Wroclaw, Poland; annapd@interia.pl (A.P.-D.); slawomir.budrewicz@umed.wroc.pl (S.B.); 9 Department of Neurology, Pomeranian Medical University in Szczecin, Unii Lubelskiej 1, 71-252 Szczecin, Poland; synaps3@wp.pl; 10 Department of Neurology, Regional Hospital, Arkonska 4, 71-455 Szczecin, Poland; anuszek-4@o2.pl; 11 Department of Neurology, 10th Military Research Hospital and Polyclinic, Powstancow Warszawy 5, 85-681 Bydgoszcz, Poland; swawrzyniak@wp.pl; 12 Department of Neurology, Copernicus Pl, M. Kopernik Hospital, Nowe Ogrody 1-6, 80-803 Gdansk, Poland; w.fryze@wp.pl; 13 Department of Public Health, Medical University of Lublin, 20-954 Lublin, Poland; marzenafurtakniczyporuk@umlub.pl

**Keywords:** relapsing-remitting type of multiple sclerosis, no evidence of disease activity (NEDA), modified Rio score (MRS), natalizumab, fingolimod

## Abstract

Background: Reliable markers of disease outcomes in multiple sclerosis (MS) would help to predict the response to treatment in patients treated with high efficacy drugs. No evidence of disease activity (NEDA) has become a treatment goal whereas the modified Rio score (MRS) predicts future suboptimal responders to treatment. The aim of our study was to identify factors that would predict poor response to treatment with natalizumab and fingolimod. Methods: In the multicenter prospective trial, 336 subjects were enrolled, initiating therapy with natalizumab (*n* = 135) or fingolimod (*n* = 201). Data on relapse rate, the expanded disability status scale, and MRI results were collected, and MRS was estimated. Results: NEDA-3 after the first year of therapy was 73.9% for natalizumab and 54.8% for fingolimod (*p* < 0.0001). Patients with MRS = 0 in the last year on platform therapy had the best NEDA-3 (71%) and patients with MRS = 3 had the worst NEDA-3 (41%) in the first year of treatment with the second-line therapy. Conclusion: We conclude that switching to the second-line therapy should occur earlier to enable better results for patients treated with natalizumab or fingolimod. The outcome on both drugs is better with better neurological conditions and lower MRS of the patient on the platform therapy.

## 1. Introduction

Multiple sclerosis (MS) is an autoimmune neurological disease that is a chronic, progressive disability and perceived as incurable. Nevertheless, there are modern and more aggressive drugs used in the treatment of relapsing-remitting MS (RR-MS) that prevent the development of disability and treat the disease with higher efficacy than others. Among many currently available compounds, fingolimod and natalizumab belong to high efficacy drugs with the longest experience in clinical practice. They are used in case of inefficacy of the first-line drugs such as interferon-beta, glatiramer acetate, teriflunomide, or dimethyl fumarate. Fingolimod (a sphingosine-receptor agonist) and natalizumab (a humanized monoclonal antibody against the cell adhesion molecule α4-integrin) are used when MS becomes highly active because the possible side effects associated with their use are more serious (including progressive multifocal leukoencephalopathy associated mainly with natalizumab use) [1]. This is the so-called escalation therapy approach in contrast to induction therapy, used from the beginning of disease onset (i.e., treatment with immunosuppressive drugs like alemtuzumab, cladribine, mitoxantrone, or autologous-hematopoietic stem cell transplantation, followed by the long-term use of maintenance therapy with disease-modifying treatment, DMT) used especially in case of rapidly evolving or severe MS (RES) [2].

No evidence of disease activity (NEDA) is a main goal of the MS treatment strategy. NEDA-3 includes the absence of relapses, no evidence of magnetic resonance (MR) activity (no new or enlarged T2 lesions or new contrast-enhancing T1 lesions), and no progression of disability (no confirmed expanded disability status scale, EDSS, worsening) in the previous 6 months. NEDA-3 reflects mainly focal inflammatory activity measures, while NEDA-4 incorporates a neurodegenerative processes indicator, i.e., progression of disability unrelated to clinical relapse. In the case of NEDA-4, lack of brain volume loss >0.4% is also added [3,4]. Another indicator of NEDA, NEDA-5 additionally includes the level of cerebrospinal fluid neurofilament light chain as a biomarker of neurodegeneration in MS [5]. There is also a term, MEDA, indicating minimal evidence of disease activity such as marginal MRI activity (one to two new T2 lesions), in the absence of relapses and contrast-enhancing lesions [6].

With the new drugs, the achievement of NEDA status lasting a few years seems to be more and more likely. The NEDA-3 for natalizumab in the AFFIRM trial was 37% [7], whereas for fingolimod in the FREEDOMS trial was 33% and NEDA-4 in the pooled FREEDOMS/FREEDOMS II population was 19.7% [8,9].

In order to achieve NEDA, it would be optimal to identify factors early on that influence the response to treatment. A clinical score that predicts the response in interferon beta-treated patients was proposed by Rio et al. and then modified by Sormani et al. [10,11]. The Rio score combines the assessment of 1 year of clinical relapses, disability progression (measured by the EDSS), and active MRI lesions [10]. The modified Rio score (MRS) includes the combination of the MRI activity and clinical relapses, as shown in Table 1 [11]. Both scores are used to identify patients with poor outcomes during subsequent years. The Rio score was also assessed in MS patients treated with natalizumab [12].

The study aimed to identify factors assessed during initial platform therapy correlating with treatment success after switching to high efficacy drugs like natalizumab and fingolimod in the first year of treatment.

## 2. Materials and Methods

### 2.1. Subjects and Study Design

Data were collected from 336 patients initiating second-line therapy, natalizumab or fingolimod, between 2013 and 2017 in ten Polish MS centers. This was a multicenter prospective trial. Patients qualified to the treatment with natalizumab or fingolimod had to fulfil the following criteria: age above 18 years old, diagnosis of RR-MS according to the 2010 revisions of the McDonald criteria of MS [13], treatment with first-line drugs during at least one year, at least two moderate relapses requiring treatment with steroids (increase in EDSS score of 1–2 points or increase of 2 points in one or two functional systems or increase of 1 point in at least 4 functional systems) or at least one severe relapse (with increase in EDSS higher than in definition of a moderate relapse) in the previous year of treatment, and at least 2 gadolinium-enhancing lesions or at least three new T2-lesions on control MRI. According to national health fund regulations, patients with the presence of antibodies against the John Cunningham virus (JCV) in their serum could not be qualified to the treatment with natalizumab. Apart from this, the choice of the second-line drug was left to the individual center. All the eligible patients were recruited.

Exclusion criteria included a clinical course different than RR-MS and RES in naïve patients whose first DMT was fingolimod or natalizumab.

Data on relapse rate and EDSS were prospectively documented at routine clinic visits performed every three months. EDSS data were collected until December 2018. An MRI was routinely performed before treatment and every year. A standardized brain MRI protocol with gadolinium was used including T2-weighted, fluid-attenuated inversion recovery (FLAIR), diffusion-weighted imaging (DWI) and pre- and postcontrast T1-weighted sequences, at magnetic field strengths of 1.5 T. Data were retrospectively collected for the purpose of this study. On the basis of MRI and relapses, MRS was estimated.

We assessed the effectiveness of treatment in two ways. First, we calculated NEDA-3 for the first year of therapy in the group of patients that started treatment with fingolimod or natalizumab. Second, we estimated NEDA-3 in the first year of therapy with the second-line drugs depending on MRS in the last year on platform therapy before switching to the second-line therapy.

### 2.2. Statistical Analysis

The results were analyzed with the use of statistical methods, including some multidimensional tests. The Shapiro–Wilk test was used to assess normal distribution. The studied characteristics with non-normal distribution as well as qualitative and quantitative data were analyzed with the use of non-parametric tests, including the Kruskal–Wallis, ANOVA, Pearson’s Chi-squared, and Mann–Whitney U tests. General descriptive statistics methods were also used. The statistically significant *p* level was set at <0.05.

## 3. Results

### 3.1. Participant Characteristics

There were 336 patients in the whole group, including 222 females (66.1%) and 114 males (33.9%). Of the 336 patients, 201 were treated with fingolimod and 135 with natalizumab. Patients were treated previously with the first-line drugs, most of them with interferons beta (82%), 16% with glatiramer acetate, and only 2% with other drugs (dimethyl fumarate and teriflunomide, because they were not refunded in Poland at the time of study). There were no dropouts in the first year of treatment (that was taken into consideration). All patients completed at least one year of therapy with fingolimod or natalizumab in order to be included into the analysis. Characteristics of patients are given in Table 2. We found no statistically significant differences between the two cohorts of patients at baseline.

### 3.2. No Evidence of Disease Activity 3 (NEDA-3) Results in Patients Treated with Natalizumab and Fingolimod

#### 3.2.1. NEDA-3 in the Group of Patients that Started Treatment with Fingolimod or Natalizumab

After the first year of therapy 71.3% of patients treated with natalizumab and 53.9% of patients treated with fingolimod attained NEDA-3 (Table 3).

#### 3.2.2. NEDA-3 in the First Year of Therapy with the Second-Line Drugs Depending on MRS in the Last Year on Platform Therapy

We assessed NEDA-3 in the first year of treatment with fingolimod and natalizumab depending on MRS one year before switching to the second-line treatment. Patients on platform therapy with MRS = 0 one year before switching to the second-line treatment in 71% attained NEDA-3 in the first year of treatment with natalizumab or fingolimod. Of the patients with MRS = 1, 62% had NEDA-3; of the patients with MRS = 2, 65% attained NEDA-3; whereas of the patients with MRS = 3, only 41% (Chi^2^ Pearson *p* = 0.05248, R Spearman *p* = 0.02743) had NEDA-3 in the first year of treatment with natalizumab or fingolimod—Table 4. There were no significant differences between patients using interferon beta or glatiramer acetate.

## 4. Discussion

Reliable markers of disease outcomes in MS would help to predict the response to treatment in patients treated with second-line drugs who may continue to have disease activity. NEDA and MEDA have become a treatment goal and outcome measure [3]. MRS is another marker that helps to detect future suboptimal responders to treatment [11].

In our study, the higher proportion of NEDA-3 in the first year was associated with natalizumab compared to fingolimod; nevertheless, for both drugs it was very good—73.9% and 54.8% respectively. In a few studies, these two drugs were compared. In the second-Line GATE study, it was found that after 12 months of treatment, ARR was more reduced by natalizumab compared to fingolimod [14]. In another study, a higher proportion of patients treated with natalizumab (55.8%) compared to fingolimod (11.6%) achieved NEDA-3, and cortical lesions status showed the higher efficacy of natalizumab versus fingolimod in suppressing disease activity in RR-MS patients [3]. In an Italian study, after two years of treatment, natalizumab proved higher efficacy in NEDA-3 (39%) in comparison to fingolimod (22%), and confirmed regression of disability was higher in the case of natalizumab (19.2% vs. 6.7%) [15]. Baroncini et al. showed higher NEDA-3 for natalizumab (70%) than fingolimod (44%) after two years of follow-up without differences in disability worsening [16]. Similarly, in the Preziosa study, the natalizumab group had a higher proportion of freedom from MRI activity (67% vs. 36% in fingolimod group) and NEDA-3 (57% vs. 28%) [17]. In a study carried out in the Polish population, more patients under natalizumab had NEDA-3 after two and four years of follow-up compared to fingolimod (66.2% and 42.1% vs. 52.1% and 29.5%, respectively) [18].

We found an association between MRS in the year preceding the switch to the second-line drugs and NEDA-3 in the first year of therapy with these drugs (i.e., natalizumab and fingolimod). Better results were achieved with MRS = 0, MRS = 1, MRS = 2—more patients had NEDA-3 later (71.4%, 62.5%, and 65.9%, respectively) comparing to MRS = 3 (only 41.2%). MRS in the year before initiation of treatment with the second-line drugs had a significant effect on disability progression, relapses, and new T2 lesions on MRI in the first year of natalizumab or fingolimod treatment. This suggests better effects of the second-line drugs in patients with a lower activity of disease in the last year on platform therapy.

This means that a better outcome could be expected in patients that have fewer relapses and fewer new demyelinating lesions on the MRI before changing to a more aggressive treatment. Neurologists should not wait too long; it is better to aggravate therapy when a patient has moderate disease activity than wait until a severe course of the disease that would lead to more relapses, disability progression, and MRI activity on more potent drugs in the future.

Raffel and co-authors found that relapses and MRS in the first year of natalizumab treatment predicted only year 1 and year 2 EDSS progression, and that effect disappeared with a longer observation period [12]. It suggested a disconnect between natalizumab’s ability to suppress the focal inflammatory activity underlying relapses and new T2 MRI lesions, and its putative effect on the progression of long-term disability [12]. In an adjusted analysis by Kapica-Topczewska et al., a higher baseline EDSS was a predictor of relapse (*p* < 0.001) and NEDA-3 failure [18].

There were no statistically significant differences between natalizumab and fingolimod regarding the probability of no relapse, depending on a MRS higher than 0.

A limitation of this analysis is the loss of patients in the follow-up period. This study had some methodological shortcomings due to its real-world evidence (RWE) nature. Follow-up MRI scans of MS patients were not always performed in daily clinical practice; therefore, the data after 5 years of study are limited. Nevertheless, this is the first report of predictive treatment response in patients with RR-MS treated with natalizumab and fingolimod after the switch from platform therapy.

## 5. Conclusions

As patients with MRS = 0 in the last year on platform therapy had the best NEDA (71%) and patients with MRS = 3 had the worst NEDA (41%) in the first year of treatment with the second-line therapy, we conclude that switching to the second-line therapy should occur earlier to enable better results for the patients treated with natalizumab or fingolimod. We should not wait too long as patients with higher MRS on interferon beta or glatiramer acetate would have a worse outcome on second-line drugs. If a patient has both radiological activity (4 or more new T2 lesions) and clinical activity (2 or more relapses), then it is far less probable that such a patient has the status of NEDA in the first year of treatment with natalizumab or fingolimod. On the other hand, if we switch a patient with some radiological activity but with only one relapse or with even better neurological status (e.g., without new demyelinating lesions or without relapses), the outcome on a new drug would be better.

Comparing the results of both drugs, we can say they both suppress disease activity (natalizumab a bit better), but the outcome on both drugs is better with a better neurological condition of the patient on platform therapy.

In conclusion, the evaluation of MRS in daily clinical practice in patients on platform therapy is useful to predict the response to natalizumab/fingolimod treatment and would be helpful to optimize MS therapy. It is recommended to switch patients with a lower MRS to achieve a better outcome.

## Figures and Tables

**Table 1:** Modified Rio score scoring criteria [11].

Criterion	Change over 1st Year	Score
MRI	≤4 new T2 lesions	0
	>4 new T2 lesions	1
Relapse	No relapses	0
	1 relapse	1
	≥2 relapses	2
Modified Rio Score = MRI criterion + relapse criterion		

**Table 2:** Baseline demographic and clinical characteristic of patients treated with fingolimod and natalizumab.

Characteristic	All Patients *n* = 336	Fingolimod	Natalizumab	*p* (Mann–Whitney U)
Age, years, mean (SD)	38.7 (10.0)	39.4 (10.5)	37.6 (9.0)	0.133
Female, *n* (%)	222 (66.1)	135 (67.16)	87 (64.4)	*p* = 0.15
Onset age, years (SD)	27.5 (9.2)	27.6 (9.8)	27.2 (8.1)	0.995
Disease duration until 1/November/2018, years (SD)	10.7 (5.4)	11.2 (5.7)	9.8 (4.7)	0.094
Mean ARR before initiation of treatment, number (SD)	2.0 (0.6)	2.0 (0.7)	2.1 (0.6)	0.206
Mean EDSS before initiation of treatment, points (SD)	3.26 (1.3)	3.3 (1.3)	3.2 (1.4)	0.598
Number of T2 lesions on MRI one year before initiation of treatment, number (SD)	19.4 (6.2)	19.3 (6.5)	19.6 (5.6)	0.455
Number of Gd+ lesions on MRI one year before initiation of treatment, number (SD)	2.3 (3.7)	2.2 (3.4)	2.5 (4.1)	0.366
Patients treated	336	201 (59.8)	135 (40.2)	

ARR, annualized relapse rate; EDSS, expanded disability status scale; Gd+, gadolinium; MRI, magnetic resonance imaging; SD, standard deviation.

**Table 3:** NEDA-3 for fingolimod and natalizumab in the first year of treatment for patients that began the treatment in 2013 and 2014.

*n* = 281		NEDA-3 in the First Year of Treatment *n* (%)	*p*
All	NEDA-3 No-NEDA	169 112	
Fingolimod	NEDA-3 No-NEDA	97 (53.9) 83 (46.1)	*p* < 0.0001
Natalizumab	NEDA-3 No-NEDA	72 (71.3) 29 (28.7)	

NEDA, no evidence of disease activity; available data from *n* = 281.

**Table 4:** Relationship between MRS on platform therapy before switching to the second-line treatment and NEDA-3 in the first year of treatment with natalizumab or fingolimod.

		MRS = 0	MRS = 1	MRS = 2	MRS = 3	Chi^2^ Pearson R Spearman
All	NEDA-3 No-NEDA-3	5 (71.4%) 2 (28.6%)	10 (62.5%) 6 (37.5%)	118 (65.9%) 61 (34.1%)	14 (41.2%) 20 (58.8%)	*p* = 0.05248 *p* = 0.02743

IFNs, interferons; MRS, modified Rio score; NEDA, no evidence of disease activity.

## Data Availability

The data presented in this study are available on request from the corresponding author. The data are not publicly available due to ethical restrictions.
